# Supplementary material for: Impact of Campylobacter spp. on the Integrity of the Porcine Gut
Source: Animals (Basel). 2021 Sep 19;11(9):2742. doi: 10.3390/ani11092742 (PMC8467837; doi:10.3390/ani11092742)
Supplement: Supplementary file 1 [file animals-11-02742-s001.zip › animals-1336452-supplementary.pdf]

Fecal sampling → Plating on CCDA agar and enrichment in Preston broth

↓ Incubation period for 48h

Evaluation of the first CCDA agar and strike out Preston enrichment on CCDA agar

↓ Incubation period for 48h

Evaluation of the CCDA agar with the Preston enrichment

Figure S1. Microbiological procedure for the detection of *Campylobacter* spp. in the fecal samples of the test animals on modified charcoal cefoperazonedeoxycholate agar plates (CCDA).

Table S1. Ingredients of the nalidixic acid stock solution (1%).

Chemicals	Amount of Substance in the Solution
Nalidixin acid	1.0 g
Sodium hydroxide (NaOH, 1 mol/l)	6.0 ml
double distilled water	fill up to 100 ml

Table S2. Ingredients of the streptomycin stock solution (1%).

Chemicals	Amount of Substance in the Solution
Streptomycin sulphate	1.0 g
double distilled water	fill up to 100 ml

Table S3. Quantity ratio for the preparation of the specific antibiotics-CCDA agar with the respective stock solution (nalidixic acid or streptomycin sulphate).

Chemicals	Amount of Substance in the Solution
CCDA selective Supplement SR0155E [1]	1 unit
Campylobacter agar base CM0739B [1]	22.75 g
double distilled water	500 ml
nalidixic acid stock solution (1%) or streptomycin stock solution (1%)	5 ml

[1] Thermo Scientific Inc., Waltham, MA, USA.

Table S4. Body temperature of the infection groups over the experimental period as mean ± standard deviation (Group 0: $n = 11$; Group 1: $n = 8$; Group 2: $n = 10$; Group 3: $n = 8$).

Infection Group	Body temperature (°C)
Group 0	39.53 ± 0.09
Group 1	39.54 ± 0.08
Group 2	39,59 ± 0.11
Group 3	39,56 ± 0.16

Table S5. ΔI_{sc} values for jejunal tissue, both basal values for the groups and the changes after addition of glucose and forskolin, the mean values and standard deviations for the respective groups (0–3) are shown (Group 0: $n = 11$; Group 1: $n = 8$; Group 2: $n = 10$; Group 3: $n = 8$).

Infection Group	$\Delta I_{sc\ basal}$ ($\mu E(cm^2h)^{-1}$)	$\Delta I_{sc\ Glucose}$ ($\mu E(cm^2h)^{-1}$)	$\Delta I_{sc\ Forskolin}$ ($\mu E(cm^2h)^{-1}$)
Group 0	0.29 ± 0.16	1.24 ± 0.84	1.99 ± 0.83
Group 1	0.19 ± 0.25	0.91 ± 0.84	1.72 ± 0.53
Group 2	0.30 ± 0.31	1.52 ± 1.23	2.57 ± 1.21
Group 3	0.20 ± 0.13	0.53 ± 0.31	1.88 ± 0.79

Table S6. ΔI_{sc} values for caecal tissue, both basal values for the groups and the changes after addition of carbachol and forskolin, Table 0. are shown (Group 0: *n* = 11; Group 1: *n* = 8; Group 2: *n* = 10; Group 3: *n* = 8).

Infection Group	$\Delta I_{sc\ basal}$ (µE(cm²h)⁻¹)	$\Delta I_{sc\ Carbachol}$ (µE(cm²h)⁻¹)	$\Delta I_{sc\ Forskolin}$ (µE(cm²h)⁻¹)
group 0	0.17 ± 0.31	0.43 ± 0.22	2.07 ± 0.96
group 1	0.37 ± 0.35	0.30 ± 0.13	1.27 ± 0.53
group 2	0.71 ± 0.47 [1]	1.12 ± 0.63 [1]	3.66 ± 1.11 [1]
group 3	0.13 ± 0.35	0.53 ± 0.16	0.63 ± 1.07

[1] These values differ significantly from those of group 0 ($p \leq 0.05$).

Table S7. ΔG_t values for jejunal epithelia, both basal values for the groups and the changes after addition of glucose and forskolin, the mean values and standard deviations for the respective groups (0–3) are shown (Group 0: *n* = 11; Group 1: *n* = 8; Group 2: *n* = 10; Group 3: *n* = 8).

Infection Group	ΔG_t basal (mS cm⁻²)	ΔG_t glucose (mS cm⁻²)	ΔG_t forskolin (mS cm⁻²)
Group 0	28.09 ± 4.50	−2.37 ± 2.43	−0.15 ± 2.55
Group 1	24.86 ± 5.70	−1.50 ± 2.16	−0.85 ± 1.93
Group 2	28.48 ± 7.52	1.12 ± 0.63	−0.60 ± 1.17
Group 3	20.98 ± 5.68	−2.05 ± 1.63	0.20 ± 1.49

Table S8. ΔG_t values for caecal epithelia, both basal values for the groups and the changes after addition of carbachol and forskolin, the mean values and standard deviations for the respective groups (0–3) are shown (Group 0: *n* = 11; Group 1: *n* = 8; Group 2: *n* = 10; Group 3: *n* = 8).

Infection Group	ΔG_t basal (mS cm⁻²)	ΔG_t carbachol (mS cm⁻²)	ΔG_t forskolin (mS cm⁻²)
Group 0	19. 83 ± 7.13	−0.83 ± 1.23	−0.21 ± 1.53
Group 1	19.80 ± 1.69	−0.14 ± 0.64	−0.36 ± 0.71
Group 2	18.88 ± 8.02	−0.29 ± 1.18	1.59 ± 1.30
Group 3	19.82 ± 7.77	−0.49 ± 0.75	0.63 ± 1.07
